# iTRAQ-based proteomic analysis of *Deinococcus radiodurans* in response to ^12^C^6+^ heavy ion irradiation

**DOI:** 10.1186/s12866-022-02676-x

**Published:** 2022-11-04

**Authors:** Yuan Gao, Naikang Li, Yanxia Zhou, Zhenpeng Zhang, Yao Zhang, Pengcheng Fan, Hangfan Zhou, Tao Zhang, Lei Chang, Huiying Gao, Yanchang Li, Xianjiang Kang, Qiong Xie, Zhitang Lyu, Ping Xu

**Affiliations:** 1 Central Laboratory of College of Horticulture, Nanjing Agricultural University, Nanjing, 210095 People’s Republic of China; 2 State Key Laboratory of Proteomics, Beijing Proteome Research Center, National Center for Protein Sciences Beijing, Research Unit of Proteomics & Research and Development of New Drug of Chinese Academy of Medical Sciences, Institute of Lifeomics, 38 Science Park Road, National Center for Protein Sciences (Beijing), Changping District, Beijing, 102206 People’s Republic of China; 3 School of Life Sciences, Institute of Life Science and Green DevelopmentHebei University and Key Laboratory of Microbial Diversity Research and Application of Hebei Province, 180 East Wusi Road, Baoding, 071002 People’s Republic of China; 4 Beijing Institute of Food Inspection and Research, Beijing Municipal Center for Food Safety Monitoring and Risk Assessment, Beijing, 102206 People’s Republic of China; 5 China Astronaut Research and Training Center, Beijing, 100094 People’s Republic of China; 6 Anhui Medical University, Hefei, 230032 People’s Republic of China; 7 Medical School of Guizhou University, Guiyang, 550025 People’s Republic of China; 8 Second Clinical Medicine Collage, Guangzhou University Chinese Medicine, Guangzhou, 510006 People’s Republic of China

**Keywords:** *Deinococcus radiodurans*, Heavy ion irradiation, Proteome, iTRAQ quantitative mass spectrometry, Antioxidation regulation

## Abstract

**Background:**

*Deinococcus radiodurans* (*D. radiodurans*) is best known for its extreme resistance to diverse environmental stress factors, including ionizing radiation (IR), ultraviolet (UV) irradiation, oxidative stress, and high temperatures. Robust DNA repair system and antioxidant system have been demonstrated to contribute to extreme resistance in *D. radiodurans.* However, practically all studies on the mechanism underlying *D. radiodurans*’s extraordinary resistance relied on the treated strain during the post-treatment recovery lag phase to identify the key elements involved. The direct gene or protein changes of *D. radiodurans* after stress have not yet been characterized.

**Results:**

In this study, we performed a proteomics profiling on *D. radiodurans* right after the heavy ion irradiation treatment, to discover the altered proteins that were quickly responsive to IR in *D. radiodurans*. Our study found that *D. radiodurans* shown exceptional resistance to ^12^C^6+^ heavy ion irradiation, in contrast to *Escherichia coli* (*E.coli)* strains. By using iTRAQ (Isobaric Tags for Relative and Absolute Quantitation)-based quantitative mass spectrometry analysis, the kinetics of proteome changes induced by various dosages of ^12^C^6+^ heavy ion irradiation were mapped. The results revealed that 452 proteins were differentially expressed under heavy ion irradiation, with the majority of proteins being upregulated, indicating the upregulation of functional categories of translation, TCA cycle (Tricarboxylic Acid cycle), and antioxidation regulation under heavy ion irradiation.

**Conclusions:**

This study shows how *D. radiodurans* reacts to exposure to ^12^C^6+^ heavy ion irradiation in terms of its overall protein expression profile. Most importantly, comparing the proteome profiling of *D. radiodurans* directly after heavy ion irradiation with research on the post-irradiation recovery phase would potentially provide a better understanding of mechanisms underlying the extreme radioresistance in *D. radiodurans*.

**Supplementary Information:**

The online version contains supplementary material available at 10.1186/s12866-022-02676-x.

## Background

In 1956, *D. radiodurans* was first isolated from canned meat that was still spoiled after being sterilized with ionizing radiation (IR) at a dose of 4 kGy [1]. Then it was found that exponential phase of *D. radiodurans* could survive even under as high as 5,000 Gy doses of gamma radiation, without losing viability or showing any signs of DNA damage-induced mutation [2]. In the decades that followed, *D. radiodurans* is well known as an extremophile, with extremely resistance to a variety of stressors including IR, oxidative stress, desiccation, and cold/heat shock [3–7].

Exposure to extreme conditions such as IR, UV (Ultraviolet) radiation and desiccation would cause an increase of reactive oxygen species (ROS), which in turn caused massive DNA damage [8, 9]. Numerous lines of research spanning more than 50 years have shown the benefits of the extremely radio-resistance of *D. radiodurans* from its super-efficient DNA damage repair system [8, 10–12]. Transcriptomic and proteomic analyses have identified several genes/proteins response to DNA repair in *D. radiodurans* under stress conditions, such as RecA, GyrA, GyrB, UvrA, UvrB, DdrA, DdrB, DdrC, DdrD and PprA [13–17]. Currently, studies show that excepting for DNA repair system, antioxidant defense system also contribute to the radio-resistance of *D. radiodurans* [18–20]. The antioxidant system of *D. radiodurans* protects the proteome from oxidative damage, allowing DNA repair system and metabolic system to function normally [21]. The antioxidant system includes the reduced endogenous respiratory chain enzymes leading to a constitutively less ROS production in *D. radiodurans* [8, 22], non-enzymatic antioxidants (such as manganese complexes [9, 18, 23]), transcriptional regulators (PprM [24] and PprI [25, 26]) and sRNA [27].

Proteins are the final functional entities of cells that regulate the cell phenotype and stress response. The latest advanced techniques in omics, particularly in proteomic study, provide a powerful tool to uncover the molecular mechanisms of extreme resistance of *D. radiodurans*. In this study, we conducted a proteomic profiling on different doses of ^12^C^6+^ heavy ion irradiation treatment of *D. radiodurans*, based on an iTRAQ-based quantitative mass spectrometry analysis. Different from the previous studies, which were carried out mainly on the post-irradiation recovery condition, our study focused on the protein change of *D. radiodurans* directly after heavy ion irradiation, which might, from another perspective, unravel the molecular mechanisms of stress response.

## Results

### Effects of ^12^C^6+^heavy ion radiation on survival of ***D. radiodurans***

**Fig. 1:**
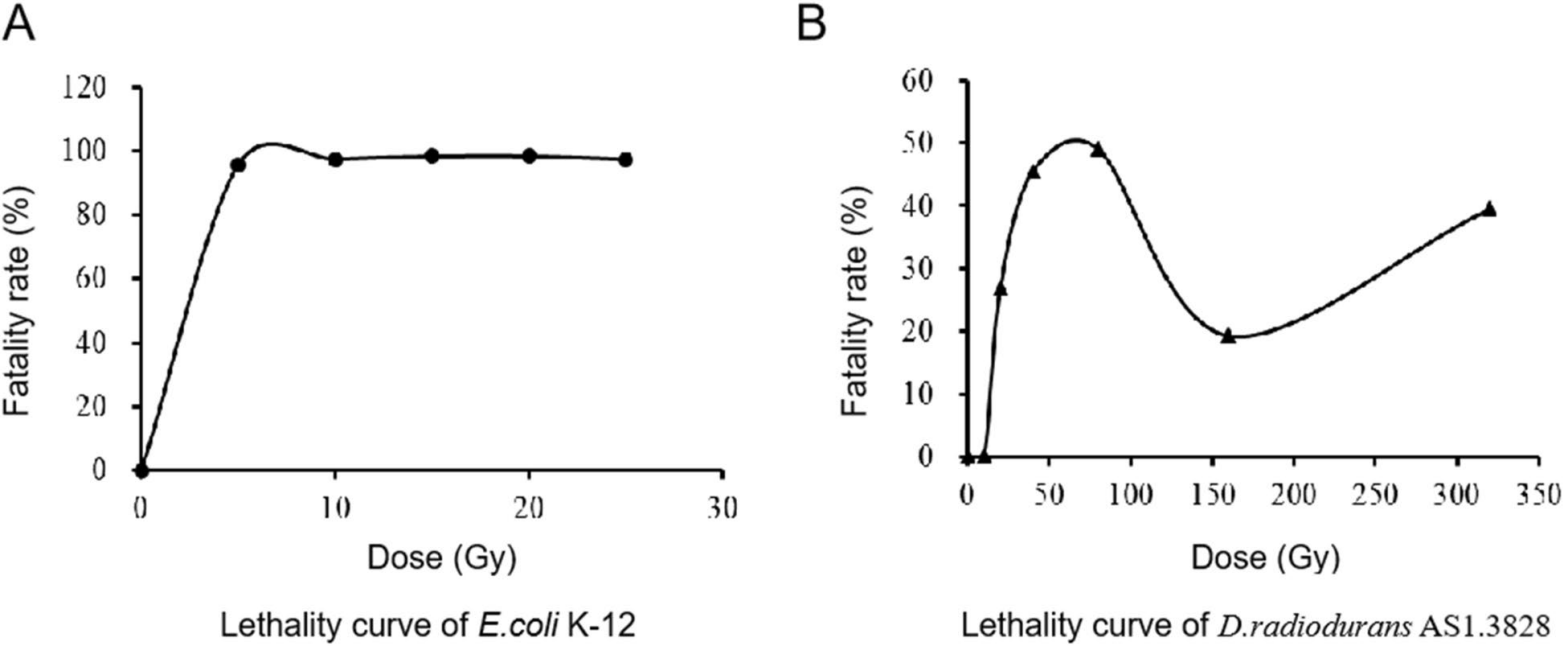
Lethality curves of *E. coli* (**A**) and *D. radiodurans* (**B**) exposed to heavy ion irradiation at different doses

Here, we tested heavy ion irradiation effect on survival of *D. radiodurans*. We used a various dose of ^12^C^6+^heavy ion to treat *D. radiodurans* and *E. coli* K-12. The nonresistant *E. coli* K-12 was used as a negative control. Exposed to a low dose of ^12^C^6+^ heavy ion irradiation, the fatality rate of *E.coli* was significantly increased, and quickly reached to saturate (up to 95.8%) in 5 Gy ^12^C^6+^ heavy ion irradiation (Fig. 1A). Compared to the previous study [28] that there was no growth of *E.coli* DH5α in the 3 kGy γ-radiation dose, our study suggested the hyper lethal effect of ^12^C^6+^ heavy ion radiation on *E.coli*. While, the mortality rate of *D. radiodurans* increased gradually with the increase of the ^12^C^6+^heavy ion dose from 0 to 80 Gy. Even at 80 Gy ^12^C^6+^ heavy ion irradiation, there was still growth for *D. radiodurans* with 49% mortality rate (Fig. 1B). Then, with the increase of the dose of ^12^C^6+^heavy ion radiation from 80 to 150 Gy, the mortality rate of *D. radiodurans* gradually decreased, low to 20%. This phenomenon is called the overkill effect, which had been reported in mammalian cells [29–31]. Previous studies have found that the cell killing effect of ionizing radiation depends upon linear energy transfer (LET) and relative biological effectiveness (RBE) that reaches a maximum at LET around 200 keV/mm, and then decreases abruptly, further declining to less than 1.0 in regard to very high LET heavy-ions [30]. The lowest mortality observed in 150 Gy compared to that in 80 and 320 Gy suggested the overkill effect in 150 Gy, with the drop of RBE in high LET.

These results suggest the extreme resistance of *D. radiodurans* to heavy ion irradiation, which is consistent with the data reported previously [9].

### Proteomics profiling of *D. radiodurans* under heavy ion irradiation

**Fig. 2:**
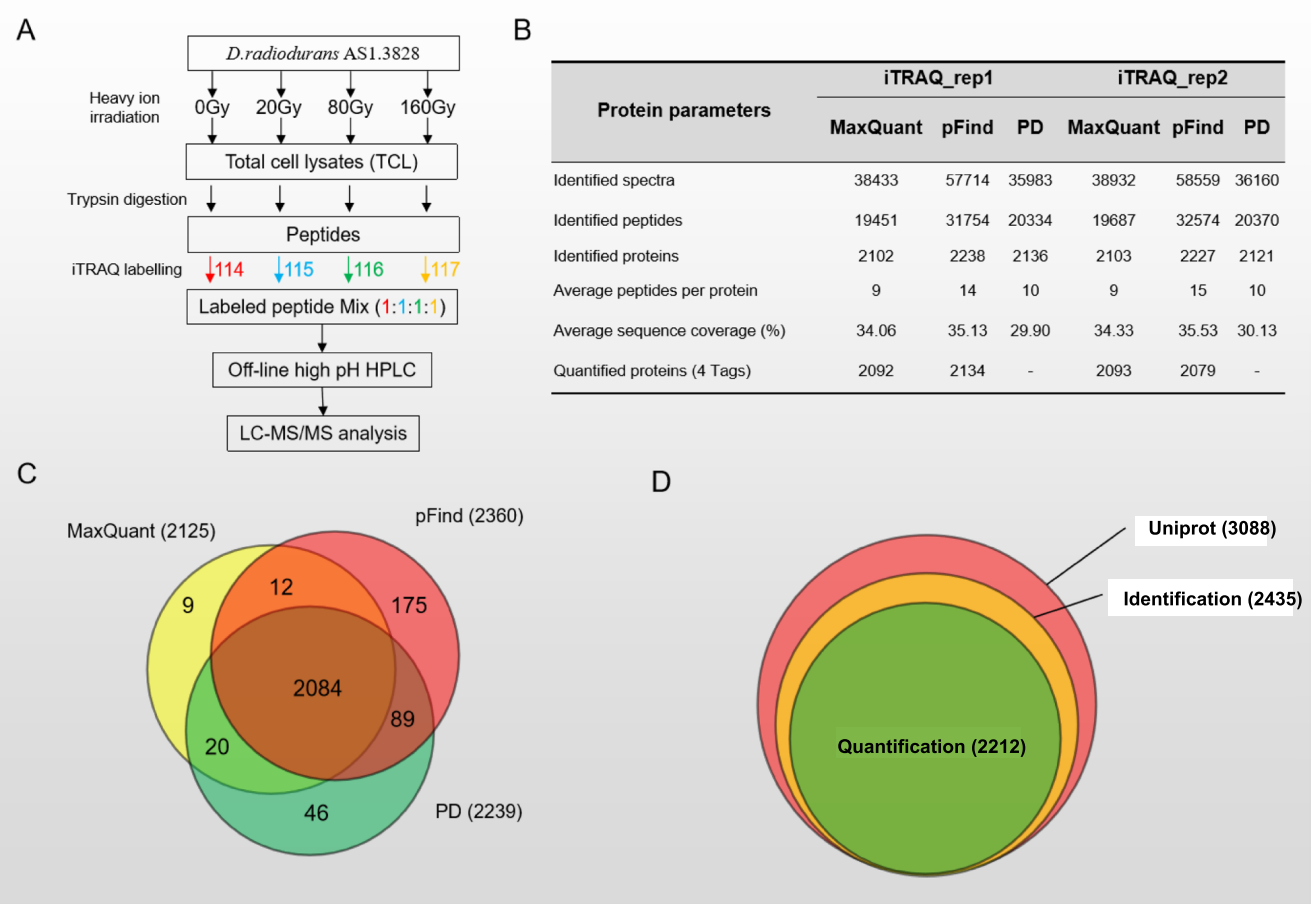
Proteomics profiling of *D. radiodurans* under heavy ion irradiation. **A**. Proteomics workflow processed by heavy ion irradiation of *D. radiodurans* AS1. 3828. **B** Summary table for proteomic identification and quantification by the three search engines. **C** Overlap in identification proteins among MaxQuant, pFind and PD. **D** Overlap of Uniprot database of *D. radiodurans*, identified proteins and quantified proteins in our dataset

To investigate the adaptive mechanism of *D. radiodurans* to heavy ion irradiation, we examined the proteomics of *D. radiodurans* at different doses of ^12^C^6+^ heavy ion irradiation by the iTRAQ quantitative approach (Fig. 2A). For this purpose, whole cell lysates of 0, 20, 80, 160 Gy ^12^C^6+^ heavy ions treated *D. radiodurans* were extracted respectively, and separated by a 10% SDS-PAGE (sodium dodecyl sulfate polyacrylamide gel electrophoresis). No significant differences were observed among these four samples, which tended to exhibit similar patterns. Clearly defined bands for high- to low-molecular weight proteins were observed in Additional file1A, indicating the high quality of whole cell lysates extracted with a similar concentration. The same amount of whole cell lysates from these four samples were loaded onto another SDS-PAGE, in-gel-digested with trypsin after separation, and tagged with iTRAQ reagents separately. The 114 iTRAQ tag was used for the 0 Gy heavy ion irradiation, 115 for 20 Gy, 116 for 80 Gy and 117 for 160 Gy sample, respectively (Fig. 2A). Differentially tagged peptides were mixed in 1:1:1:1 and analyzed by an LC–MS/MS (Liquid Chromatograph Mass Spectrometer) (Additional file 1). To assess the technical variation, we divided the same proteins obtained from 0/20/80/160 Gy irradiated samples in two identical portions and used in duplicate, which allowed us to determine the variation introduced during in-gel digestion, labelling and subsequent analysis.

To achieve deep coverage and high confidence in proteome profiling, we used three search engines including MaxQuant (1.5.5.1), pFind (version 3.1) and Proteome Discoverer (v2.4) (PD) to analyze the iTRAQ MS raw data. The number of proteins identified in each search tool was all over 2000 (Fig. 2B). A total of 2435 proteins were identified (Fig. 2C), covering almost 80% of the *D. radiodurans* proteome annotated in Uniprot (version UP000076988), and almost 91% of identified proteins were also quantified (Fig. 2D, Additional file 2). The average sequence coverage for all identified proteins achieved 33.18%, which supports the high reliability of protein identification in this study.

By comparing the proteins identified by the three search engines, the shared number of proteins reaches 2084, which occupies 98%, 88% and 93% of all the proteins identified from MaxQuant, pFind and PD, respectively (Fig. 2C). The uniformity of identification of the different software is quite high. Therefore, we selected the results of the classic MaxQuant software for further quantitative data analyses.

First, we found no obvious difference between the technical repetitions performed on the 0/20/80/160 Gy irradiated samples. The SD (standard deviation) values for two replicates of 20/0, 80/0 and 160/0 Gy were 0.018, 0.016, 0.021, 0.02, 0.021, 0.02, respectively (Additional file 3), and the corresponding R^2^ values of 0.809, 0.677 and 0.784 (Additional file 4), suggesting that our results were highly reproducible and that the experimental procedures were well controlled.

### Global analysis of the differentially expressed proteins in *D. radiodurans* under heavy ion irradiation

**Fig. 3:**
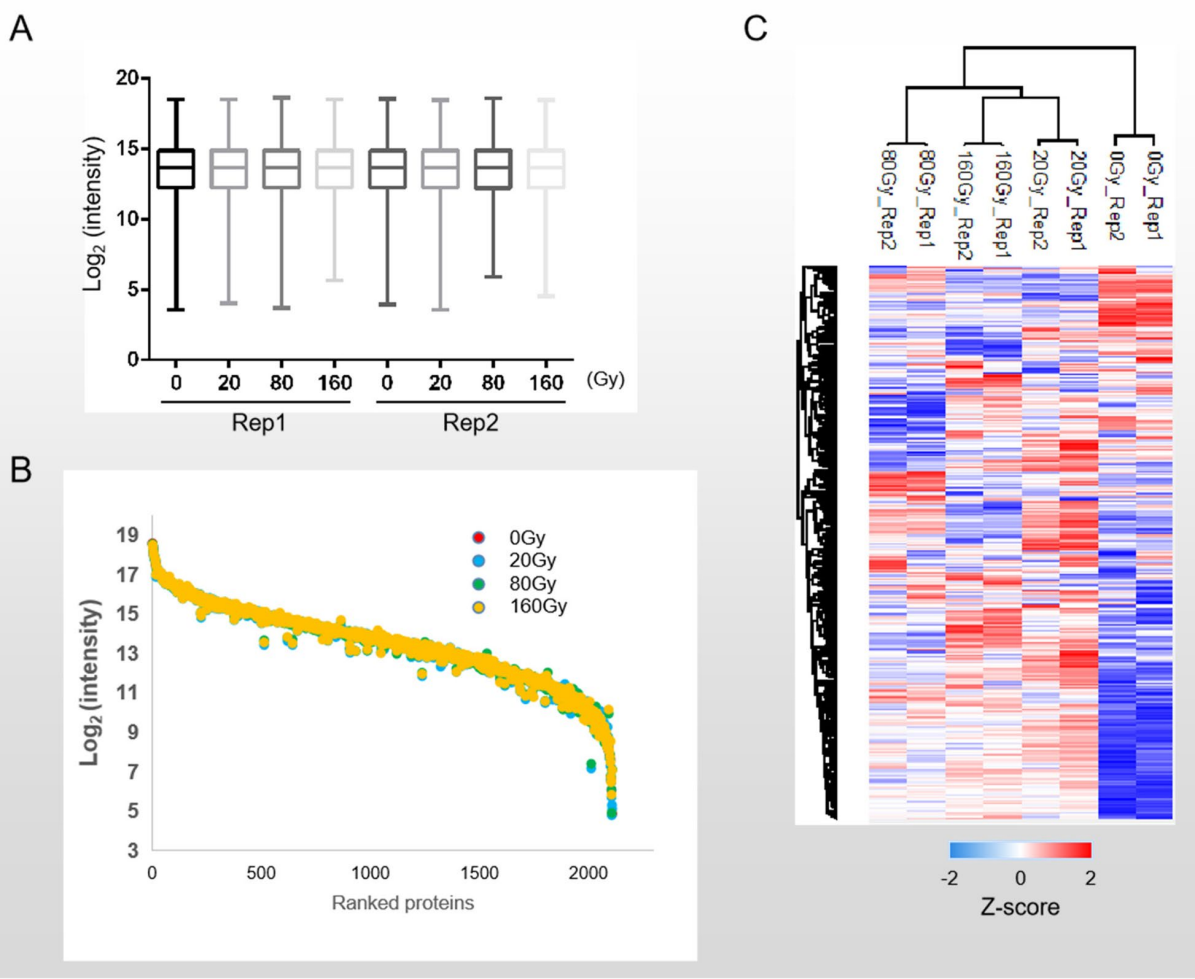
Quantification stability of heavy ion treated proteomics of *D. radiodurans.* **A**. Boxplot to show quantification and median of different treatment assays. **B**. Dotplot for range of protein intensity among 4 kinds of treatment assays. C. Heatmap to show global proteome change in 4 kinds of treatment assays

Boxplot showed the quantification and median of each assay. There was no significant difference among eight assays, suggesting the high stability of our dataset (Fig. 3A). We discovered a wide dynamic range of protein abundance among various treatment samples, spanning around 14-orders of magnitude (Fig. 3B), indicating the deep coverage of our proteomics dataset. The global proteome changes of the 2435 proteins commonly identified under varied dosages of ^12^C^6+^heavy ions demonstrated the dynamic shift in protein expression after heavy ion irradiation (Fig. 3C).

**Fig. 4:**
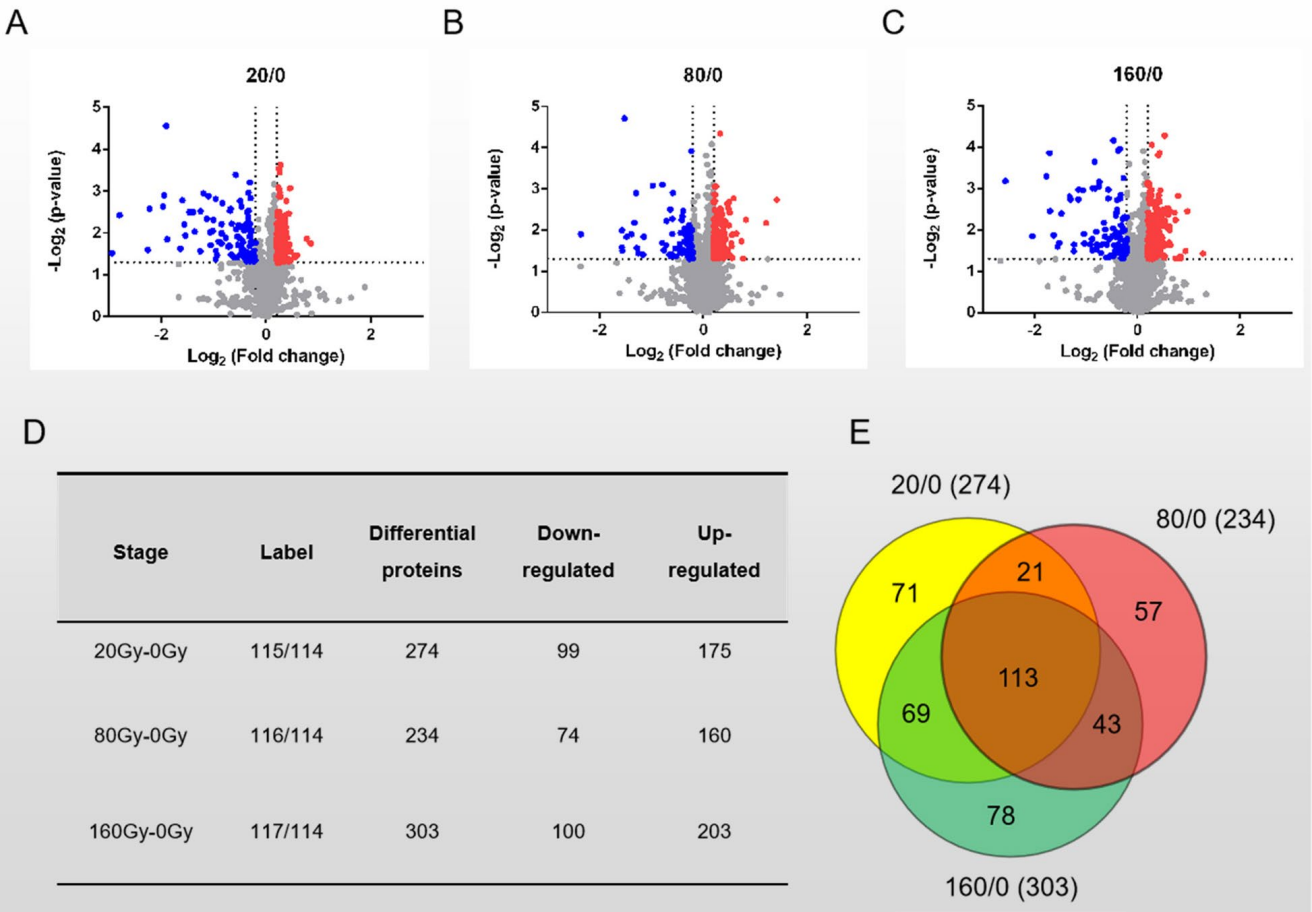
Comparison of the differentially expressed proteins in 20/80/160 Gy heavy ion irradiation condition. **A-C**. Volcanic plot to show the differential proteins screened by Log_2_ (FC) > 0.2, ***p***-value ≤ 0.05. 20 Gy, 80 Gy and 160 Gy were all compared with 0 Gy. **D**. Summary of differential proteins across each group. **E**. Protein overlap between each group’s differentially expressed proteins

**Fig. 5:**
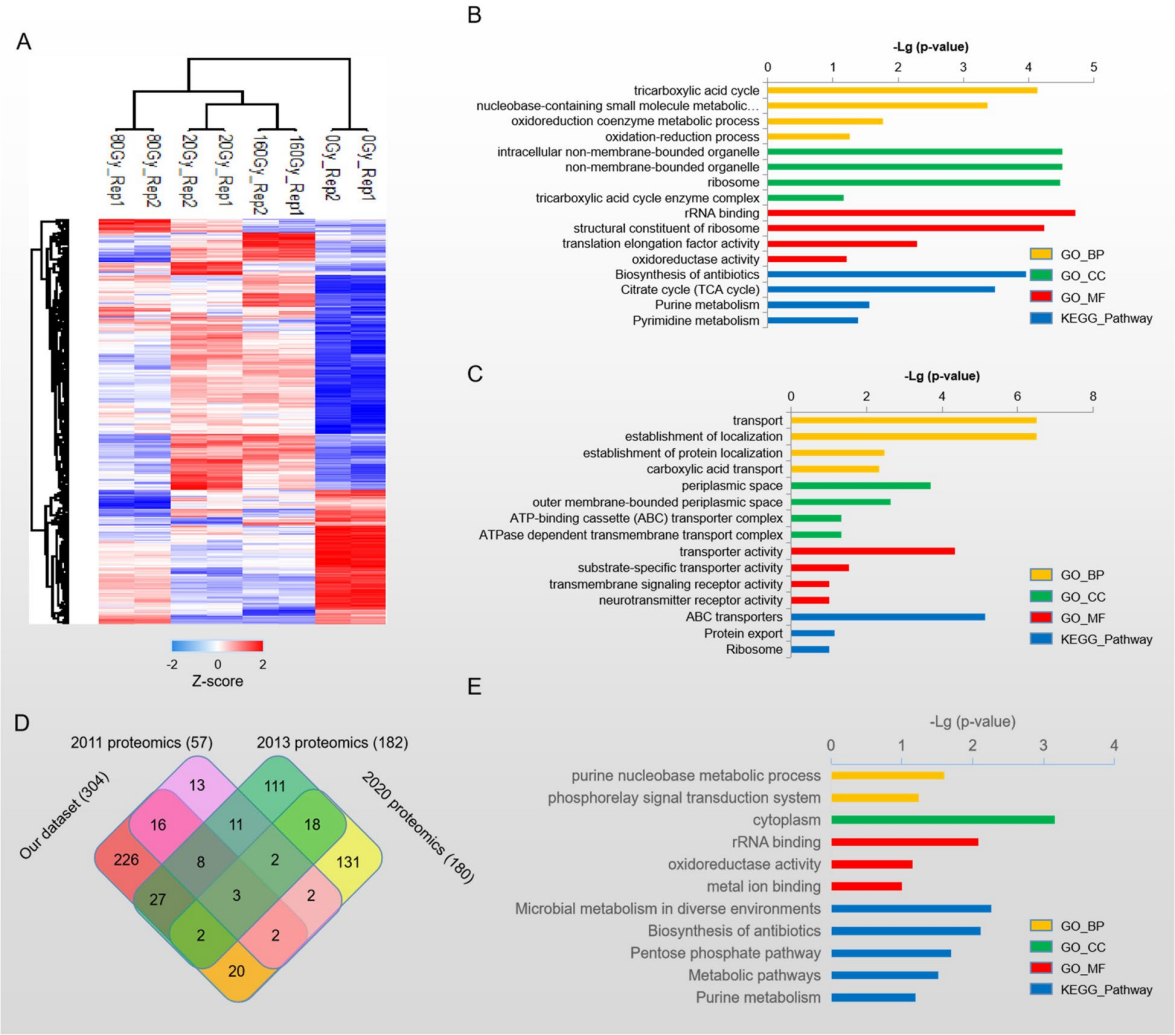
Analysis of differential expressed proteins in 20/80/160 Gy. **A**. Heatmap illustrating the relationship between 452 differential proteins across different assays. **B**. KEGG and GO enrichment analysis of 304 upregulated proteins. **C**. KEGG and GO enrichment analysis of 148 downregulated proteins. **D**. A comparison of the upregulated proteins between our dataset and the 2011 gamma radiation dataset, the 2013 nucleoid proteomics, and the 2020 ROS proteomics. **E**. KEGG and GO enrichment analysis of 226 upregulated proteins unique identified in our dataset

Based on the criteria of *p*-value < 0.05 and Log_2_ (Fold change) ≥ 0.2 (Fig. 4A-C), 274, 234 and 303 proteins were selected as differentially expressed proteins in 20/0, 80/0 and 160/0 Gy respectively. Among the identified differentially expressed proteins, 175 were upregulated and 99 were downregulated in 20 Gy irradiation, 160 were upregulated and 74 were downregulated in 80 Gy irradiation, 203 were upregulated and 100 were downregulated in 160 Gy irradiation (Fig. 4D). Venn Diagram showed 113 constantly changing proteins identified at different doses of heavy ion irradiation, including 74 upregulated proteins, 39 downregulated proteins (Fig. 4E). The molecular interactions of 113 differentially expressed proteins were predicted using STRING v11.5. The analysis produced a network with a medium clustering coefficient of 0.414, suggesting that 113 differentially expressed proteins were also belonging to a community which may be involved in similar types of function(s). STRING analysis showed that 113 differentially expressed proteins were enriched into 3 clusters (Additional file 5), two of which were known to be involved in translation and carbon metabolism. Thus, this interaction analysis indicated the translation and carbon metabolism pathway were regulated immediately in response to heavy ion irradiation. In total, 452 proteins were selected as differentially expressed proteins under ^12^C^6+^ heavy ion treatment, and majority of them were upregulated (Fig. 5A, Additional file 2). Heat map also demonstrated greater proteome consistency between samples treated with 20 Gy and 160 Gy doses compared to those treated with 80 Gy doses. This result was consistent with the cell growth under different dosages of treatment (Fig. 1B), where *D. radiodurans* demonstrated the highest fatality rate (up to 50%) under 80 Gy dosage treatment and a comparable lower fatality rate under either 20 Gy or 160 Gy dosage treatment. These results further revealed that differentially expressed proteins identified in our dataset were regulated by exposure to heavy ions, reflecting the dynamic cellular protein expression under heavy ion treatment. On the other hand, our proteomics dataset also revealed DdrD, PprI and SsB, which had been reported as important regulators involved in *Deinococcus* cells recovering from the impacts of IR [14, 32–34] (Additional file 2). According to the published results, all three of these proteins were considerably upregulated under heavy ion irradiation, further demonstrating the validity of our proteomic dataset.

We conducted GO and KEGG pathway enrichment analysis to better understand the biological processes and pathways that the differentially expressed proteins may be engaged in. Among, the upregulated proteins (304) were primarily enriched in TCA, oxidation–reduction process and translation regulation, while the downregulated proteins (148) were mostly involved in transportation regulation (Fig. 5B&C). These results were in line with earlier studies, according to which oxidative regulation, translation, TCA cycle had been reported to be elevated in response to oxidative stress [35], gamma irradiation [36] or high vacuum exposure [37], whereas ABC transporter regulation was downregulated [35].

Here we took a detailed analysis on 304 upregulated proteins in an effort to better understand the mechanisms underlying IR resistance in *D. radiodurans*. 17 proteins were commonly identified in at least 3 datasets when comparing upregulated proteins identified in our dataset with previous reported upregulated proteins under stress condition [36, 38, 39] (Fig. 5D). Based on their respective roles, these 17 proteins could be categorized into a number of groups. Group 1 includes the translation regulation-related proteins Q9RY52 (RpsF), Q9RY49 (RplI), Q9RXJ3 (RpsQ), and Q9RST0 (RplL). Group 2 includes GPT binding-related proteins Q9RWN5 (FtsZ), Q9RXK5 (FusA) and Q9RV32 (BipA). Proteins Q9RWQ9 (GroEL) and Q9RY23 (DnaK), which control unfolded protein binding, are members of group 3. Group 4 consists of the crucial proteins Q9RT53 (GyrA) and Q9RY51 (SsB), involved in DNA repair. Group 5 includes proteins Q9RTU7 (Oligopeptidase A), Q9RUP1 (Glyceraldehyde-3-phosphate dehydrogenase), Q9RU54 (Isocitrate dehydrogenase), Q9RTN7 (Aconitate hydratase A), and Q9RR60 (Enolase), which are enzymes involved in either peptide or energy metabolism. The last one protein is Q9RWM2, an uncharacterized protein. The consistent upregulation of translation, chaperonin, DNA repair and glycolysis pathway in *D. radiodurans* under different stresses points to the importance of these pathways in the stress response of *D. radiodurans*.

On the other hand, 226 unique upregulated proteins were identified in our study (Fig. 5D). Our study’s 226 unique upregulated proteins were significantly enriched in biological processes and molecular functions related to rRNA binding, oxidative regulation, and metabolism, according to GO analysis (Fig. 5E). Ten proteins, including seven oxidoreductase enzymes, were found in Table1, showing that *D. radiodurans* might utilizes numerous oxidoreductase enzymes to protect proteins or nucleic acids from increased reactive oxygen species in response to heavy ion stress. The detailed function of these oxidoreductase enzymes is worth of further exploration.

**Table 1:** Protein list of oxidoreductase activity pathway

Protein ID	Protein	Function/pathway	Differential Group
Q9RW23	Bacterioferritin comigratory protein	Hydrogen peroxide resistsnce	20/0
Q9RX79	Cytochrome B6	electron transfer activity	20/0,160/0
Q9RYN6	Flavin monoamine oxidase-related protein	oxidoreductase activity	20/0,80/0,160/0
Q9RV83	General stress protein 26	NA	20/0
Q9RYV6	Oxidoreductase, short-chain dehydrogenase/reductase family	oxidoreductase activity	80/0,160/0
Q9RT75	Oxidoreductase	D-threo-aldose 1- dehydrogenase activity	20/0
Q9RWD9	Oxidoreductase, short-chain dehydrogenase/reductase family	oxidoreductase activity	20/0,80/0,160/0
Q9RYW1	Oxidoreductase, short-chain dehydrogenase/reductase family	oxidoreductase activity	20/0,80/0
Q9RYF4	Oxidoreductase, short-chain dehydrogenase/reductase family	oxidoreductase activity	20/0,80/0,160/0
Q9RYU4	NAD(P)H dehydrogenase	NA	20/0,160/0

## Discussion

In this study we used iTRAQ-based quantitative mass spectrometry strategy to profile the cellular protein expression change of *D. radiodurans* under ^12^C^6+^ heavy ion irradiation in an effort to uncover the mechanism of its extremely high radiation resistance. To our knowledge, our quantitative dataset-which revealed direct broad-scale proteome characteristics of *D. radiodurans* under heavy ion irradiation-is the first large-scale quantitative proteomics investigation on *D. radiodurans* against high dosage of heavy ion irradiation to date. The majority of the proteins in our proteomics dataset that changed dynamically after being exposed to heavy ions were upregulated (Fig. 3C). The IR resistance of *D. radiodurans* may be attributed to the upregulated proteins under heavy ion irradiation.

Three hundred four upregulated proteins and 148 downregulated proteins, totaling 452 differentially expressed proteins under heavy ion irradiation, were identified in our proteomics dataset (Fig. 5A). To our knowledge, this is, to date, the largest differentially changed protein dataset identified in *D. radiodurans*. Among the 304 upregulated proteins, 78 proteins were identified in common in the other three published proteomics datasets (Fig. 5D), including SsB, DdrD and PprI, which were known to be associated with IR resistance. These results further indicate the reliability of our proteomics dataset. In addition, 226 upregulated proteins were unique identified in our study, accounting for 74.3% of all differentially expressed proteins in our dataset (Fig. 5D). These upregulated proteins were predominantly components of cytoplasm, and involved in biological pathways related to rRNA binding, oxidative regulation, the biosynthesis of antibiotics, biosynthesis of amino acids and secondary metabolites, and other cellular metabolism regulation. Among proteins related to antioxidation regulation, seven are oxidoreductase, while one protein (Q9RW23) is functionally involved in antioxidation regulation. The remaining two proteins are Q9RX79, which is involved in the regulation of elector transfer, and Q9RV83 (which is a general stress protein). Our result further suggests the crucial role of antioxidation regulation in the IR resistance of *D. radiodurans*.

In our study, 304 upregulated proteins in response to heavy ion irradiation were predominantly enriched in TCA, oxidative regulation, translation, and transportation (Additional file 6). A previous proteomic analysis performed by Ott et al*.* [40] revealed proteins induced by high vacuum in *D. radiodurans* were significantly enriched in TCA cycle, nucleotide excision repair, aminoacyl-tRNA biosynthesis, and microbial metabolism in diverse environments. The results of a different proteomics analysis conducted by Basu et al*.* [36] showed that gamma radiation-inducible proteins corresponded to the key functional categories of DNA repair, oxidative stress response, protein translation/folding, and general housekeeping. According to Xue et al. [41] ‘s transcriptome investigation of *D. radiodurans*, the genes activated by heat stress were primarily involved in stress and stimulus, proteolysis, oxidation–reduction processes, DNA repair, and other biological processes. These results indicate that certain biological functions, such as DNA repair, antioxidative regulation and cellular metabolism, all of which are detected in diverse datasets, are very crucial for *D. radiodurans* to adapt to a variety of stresses. Additionally, it indicates that *D. radiodurans* may utilize the same set of gene regulation mechanisms in response to various stimuli in order to develop stress resistance.

Recently, *D. radiodurans* has also been used for the microbial production of the lycopene [42] and xanthophylls [43], which have drawn significant interest as ingredients in natural food due to their association with beauty and health. Our proteome analysis of *D. radiodurans*, along with additional metabolomics research, might reveal novel potential synthesis pathways for intriguing substances, paving the way for future uses of extremophilic microorganisms in the health-care and nutritional industries.

In conclusion, our study provides a comprehensive understanding of the cellular proteome changes that occurred in *D. radiodurans* as a result of heavy ion irradiation, which will help us better understand the radiation resistance of this species. These heavy ion-responsive proteins, are primarily involved in translation, TCA cycle, antioxidation and cellular metabolism. Further functional investigations of these proteins, particularly to these newly identified antioxidation related proteins, will enhance our understanding of the regulatory mechanism behind the extraordinary radiation resistance of *D. radiodurans*.

## Methods

### Strain and culture conditions

*D. radiodurans* CGMCC1.3828, which originated from *D. radiodurans* R1, was purchased from the China General Microbiological Culture Collection Center (CGMCC, Beijing, China). *D. radiodurans* CGMCC1.3828 was cultured in Corynebacterium medium (1% tryptone, 0.5% yeast extract, 0.5% glucose, and 0.5% NaCl) at 30 °C with shaking at 200 r/min or on Corynebacterium plates, while *E. coli* K-12 was cultured in Luria–Bertani (LB) medium at 37℃.

### Pretreatment for irradiation

*D. radiodurans* CGMCC1.3828 was cultured overnight in Corynebacterium medium at 30 °C and grown in fresh Corynebacterium medium up to mid-log phase (OD_600_ = 9). For the purpose of irradiation treatment, cell suspensions were centrifuged at 10,000 r/min for 3 min, washed three times with 0.9% saline, and then resuspended in 0.1 M Na_2_HPO_4_-NaH_2_PO_4_ (pH 7.0) buffer. *E. coli* K-12 was cultured in LB medium at 37 °C to mid-log phase (OD_600_ = 2). *E. coli* cells were collected after centrifuging at 10,000 r/min for 3 min, washed three times with 0.9% saline, and then resuspended by 0.1 M Na_2_HPO_4_-NaH_2_PO_4_ (pH 7.0) buffer for irradiation treatment.

### Irradiation treatment and screening process

^12^C^6+^ heavy ion irradiation was conducted by the Heavy-Ion Research Facility in Lanzhou (HIRFL), Institute of Modern Physics, Chinese Academy of Sciences. *D. radiodurans* CGMCC1.3828 suspensions (OD_600_ = 1) and *E. coli* K-12 suspensions (OD_600_ = 1) were irradiated with a carbon ion beam at 32 keV/μm at a rate of 30 Gy/min. Seven different doses were administered to *D. radiodurans* CGMCC1.3828, including 0、10、20、40、80、160 and 320 Gy. Six different doses were used to treat *E. coli* K-12, including 0、5、10、15、20 and 25 Gy. With the exception of irradiation, unirradiated cells received the same treatment as irradiated cells. After irradiation, *D. radiodurans* cell suspensions were diluted to 10^–4^ and 10^–5^, and *E. coli* cell suspensions were diluted to 10^–6^ and 10^–7^. 100 μL of the dilution were spread on the Corynebacterium agar plate and LB plate, which were then cultivated at 30 °C and 37 °C, respectively. The mortality rate was expressed as the percentage of the decreased number of colonies in the treated samples compared with that of the untreated sample, which was used as a control. Remaining cell suspensions were collected at 13,300 r/min for 5 min, and cell pellets were stored at -80 °C.

### Protein extraction, digestion and TMT labelling

Heavy ion irradiated cell pellets at 0、20、80 and 160 Gy stored at -80 °C were used for protein extraction. Cells were suspended in a lysis buffer (50 mM NH_4_HCO_3_, 8 M urea, 5 mM IAA, 1xEDTA-free protease inhibitor cocktail) and disrupted using ultrasonic crushing at 30% ultrasonic power. The supernatants were collected after centrifugation at 13,300 r/min at 4 °C for 10 min, and the total cell protein was then quantified using a previously described gel-assisted method [44]. The gel was imaged and the image was analyzed by Scion Image software (4.0.3.2) (National Institutes of Health, Bethesda, MD, USA).

An equal amount of total cellular lysates (120 μg) from 0、20、80 and 160 Gy irradiated *D. radiodurans* was processed. These protein samples were reduced by 5 mM dithiothreitol, alkylated by 20 mM of IAA, precleaned by short SDS PAGE (10%, 1 cm), and digested in-gel by 12.5 ng/μL trypsin (Meizhiyuan, Beijing, China) at 37 °C for 12 h [45]. As directed by manufacturer, iTRAQ reagents (Sigma-Aldrich, St. Louis, USA) were used to label tryptic peptides. Briefly, the same amount of peptides from each group were labeled with the distinct iTRAQ tags (Fig. 2A). The reaction was quenched by adding 8 μL of 5% hydroxylamine. It was then mixed and dried in a vacuum dryer (CentriVap; LABCONCO, Kansas City, USA).

### LC–MS/MS analysis by Q-Exactive and sequence database searching

The iTRAQ-labeled peptides were desalted by Sep-Pak tC18 cartridges (Waters, Milford, USA) after being resolved with 2% acetonitrile and 0.1% trifluoroacetic acid. The desalted peptides were further separated by a high-pH reverse-phase HPLC system equipped with an XBridge Peptide BEH C18 analytical column (5 μm, 4.6 × 250 mm, Waters, Milford, USA). Simply, buffer A (2% acetonitrile, ACN in dd H_2_O, pH 10.0) was used to equilibrate the HPLC column. The loaded peptides were eluted by the linear elution method at a gradient of 2% to 80% buffer B (98% ACN, pH 10.0) in 80 min at a flow rate of 1 mL/min. The eluted samples were collected every minute and then pooled into nine fractions for liquid chromatography with tandem mass spectrometry (LC–MS/MS) analysis after being dried following the similar procedure described previously [46]. In brief, the resuspended samples were analyzed using a Q-Exactive HF mass spectrometer (Thermo Fisher Scientific, Waltham, USA). Briefly, samples were loaded onto a self-packed capillary column (75 μm i.d. × 50 cm, 3.0 μm C18) and eluted with a 60-min linear gradient, in which solvent B was incrementally increased as follows: 0% to 5% for 6 min, 5% to 10% for 2 min, 10% to 35% for 47 min, 35% to 80% for 3 min, and held at 80% for 2 min. Full MS scans were performed over a m/z range of 300 to 1,600 at a resolution of 30,000. The maximum injection time (MIT) was set to 25 ms, and the automatic gain control (AGC) was set to 1.0 × 10^6^. For MS/MS scans, the 20 most intense peptide ions with charge states > 1 were subjected to fragmentation via higher energy collision-induced dissociation (AGC, 5000; MIT 25 ms). Dynamic exclusion was set at 30 s.

The acquired raw files were searched against a composite target/decoy database using MaxQuant (v1.5.5.1), pFind (v3.1) and Proteome Discoverer (v2.4) to determine the false discovery rate (FDR). The protein database used for MS/MS searches for *D. radiodurans* AS1.3828 was downloaded from Uniprot (http://www.uniprot.org/), including 3,088 protein sequence. The search parameters included trypsin as a proteolytic enzyme, with two missed cleavages allowed. Methionine oxidation was set as a dynamic modification, whereas cysteine carbamidomethylation, and iTRAQ 4 plexes modification at the peptide N-terminus and lysine were set as fixed modifications.

The protein identification FDR was set at 1% using a target–decoy search strategy. MaxQuant (v1.5.5.1) and Panda (v1.2.2) were used to quantified the proteins. The different expression analysis was performed using unique + razor peptides, with quantified peptides ≥ 2. The MaxQuant data was used to the differential expression analyses which was used unique + razor peptides with quantified peptides ≥ 2.

### Bioinformatics analysis

Differentially expressed proteins (DEPs) that were identified in this study were subjected to functional analyses using the DAVID online platform, including biological process (BP), and molecular function (MF) analyses [47]. Enriched pathways were obtained by KEGG database [48, 49]. Protein interaction analysis was conducted using the STRING database [50].

## Supplementary Information

**Additional file 1.** Preparation for iTRAQ quantitative proteomics study.

**Additional file 2**. Protein identification and quantification of Deinococcus radiodurans.

**Additional file 3.** The Gaussian fitting curve of log2 ratio of the intensities of two replicates of 20/0 (A), 80/0 (B) and 160/0 (C). The red and blue curves represent the experimental and Gaussian fitting curve, respectively.

**Additional file 4.** High correlation between the two technical replicates. The scatterplot of two technical replicates treated with 20/80/160 Gy heavy ion irradiation.

**Additional file 5.** String analysis of 113 overlapped differential proteins in our dataset.

**Additional file 6. **KEGG and GO enrichment analysis of 452 differential proteins in our dataset.

**Additional file 7:** **Supplementary file1.** Preparation for iTRAQ quantitative proteomics study, related to additional file 1

## Data Availability

The mass spectrometry proteomics data were deposited in the Integrated Proteome Resources database (https://www.iprox.cn/) with the dataset identifier IPX0004145000.

## Abbreviations

*E.coli*: *Escherichia coli*
*D. radiodurans*: *Deinococcus radiodurans*
iTRAQ: Isobaric tags for relative and absolute quantitation
TCA cycle: Tricarboxylic acid cycle
ROS: Reactive oxygen species
SDS-PAGE: Sodium dodecyl sulfate polyacrylamide gel electrophoresis
LC–MS: Liquid chromatograph mass spectrometer
PD: Proteome discoverer
GO: Gene ontology
KEGG: Kyoto encyclopedia of genes and genomes
DEPs: Differentially expressed proteins
BP: Biological process
MF: Molecular function
