# Supplementary material for: iTRAQ-based proteomic analysis of Deinococcus radiodurans in response to 12C6+ heavy ion irradiation
Source: BMC Microbiol. 2022 Nov 4;22:264. doi: 10.1186/s12866-022-02676-x (PMC9635210; doi:10.1186/s12866-022-02676-x)
Supplement: Supplementary file 1 — Additional file 1. Preparation for iTRAQ quantitative proteomics study. [file 12866_2022_2676_MOESM1_ESM.pptx]

## Slide 1
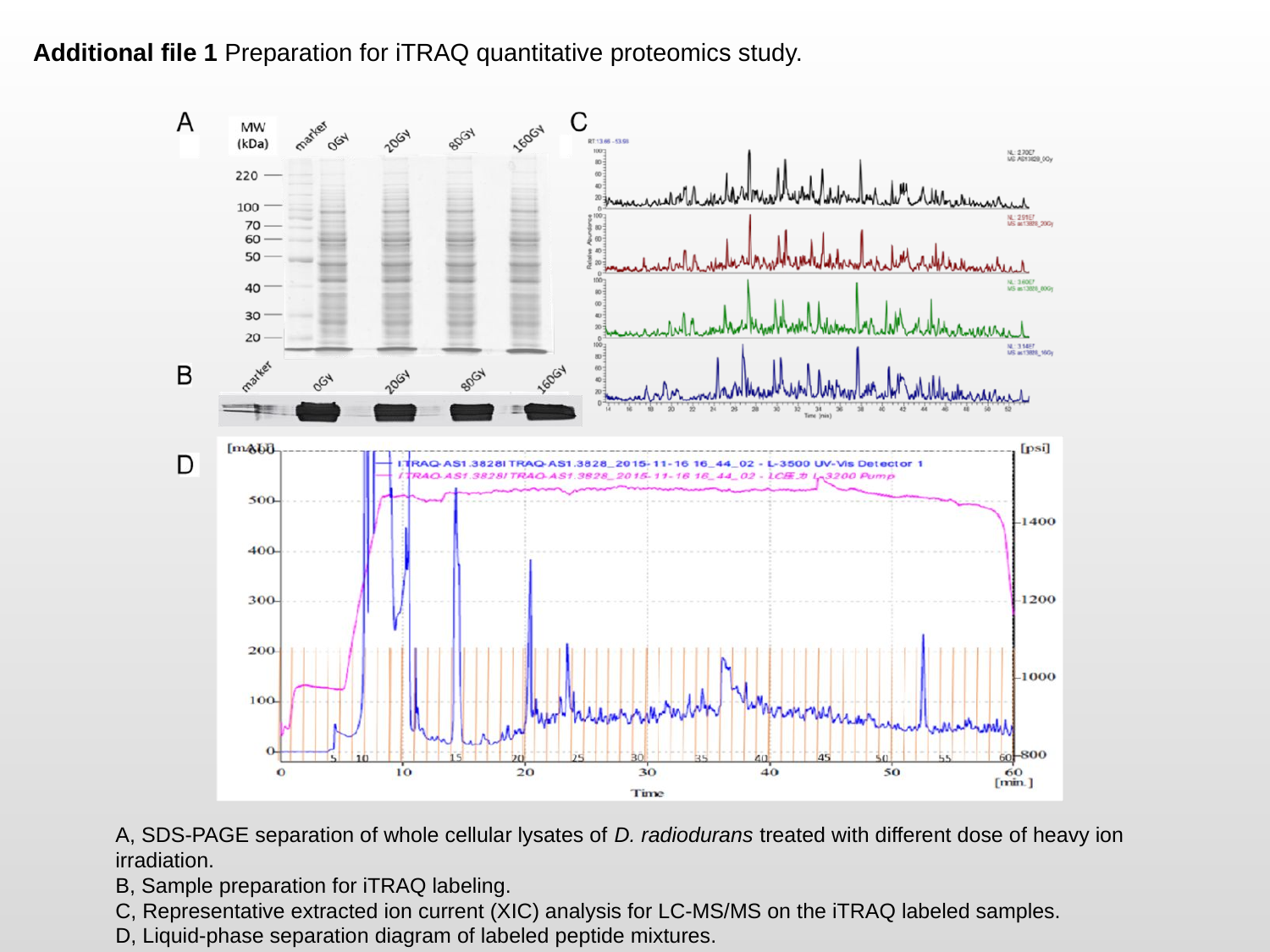

Additional file 1 Preparation for iTRAQ quantitative proteomics study.
A, SDS-PAGE separation of whole cellular lysates of D. radiodurans treated with different dose of heavy ion irradiation.
B, Sample preparation for iTRAQ labeling.
C, Representative extracted ion current (XIC) analysis for LC-MS/MS on the iTRAQ labeled samples.
D, Liquid-phase separation diagram of labeled peptide mixtures.
