# Supplementary material for: iTRAQ-based proteomic analysis of Deinococcus radiodurans in response to 12C6+ heavy ion irradiation
Source: BMC Microbiol. 2022 Nov 4;22:264. doi: 10.1186/s12866-022-02676-x (PMC9635210; doi:10.1186/s12866-022-02676-x)
Supplement: Supplementary file 3 — Additional file 3. The Gaussian fitting curve of log2 ratio of the intensities of two replicates of 20/0 (A), 80/0 (B) and 160/0 (C). The red and blue curves represent the experimental and Gaussian fitting curve, respectively. [file 12866_2022_2676_MOESM3_ESM.pptx]

## Slide 1
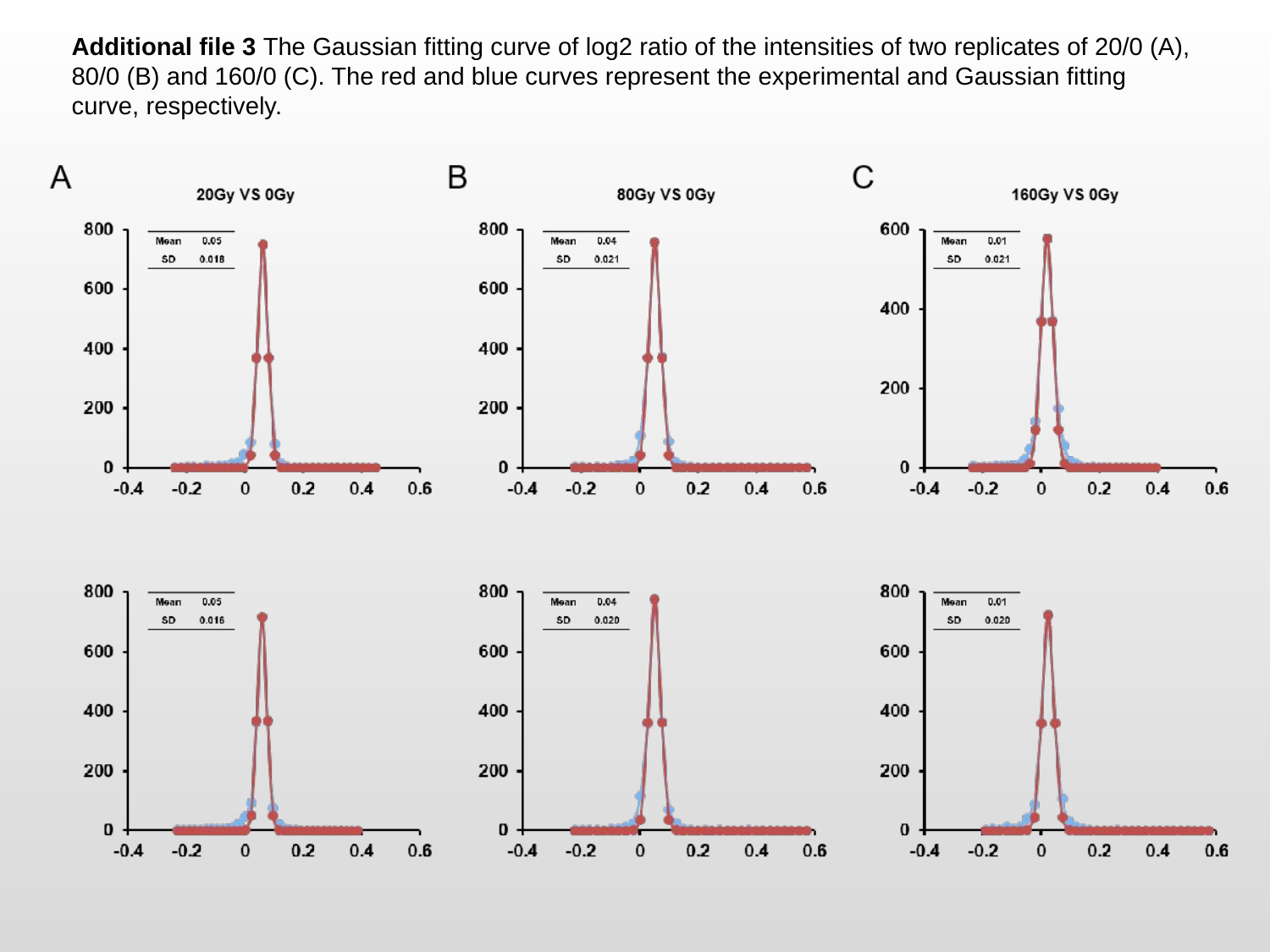

Additional file 3 The Gaussian fitting curve of log2 ratio of the intensities of two replicates of 20/0 (A), 80/0 (B) and 160/0 (C). The red and blue curves represent the experimental and Gaussian fitting curve, respectively.
