# Supplementary figures and images for: iTRAQ-based proteomic analysis of Deinococcus radiodurans in response to 12C6+ heavy ion irradiation
Source: BMC Microbiol. 2022 Nov 4;22:264. doi: 10.1186/s12866-022-02676-x (PMC9635210; doi:10.1186/s12866-022-02676-x)

## Slide 1
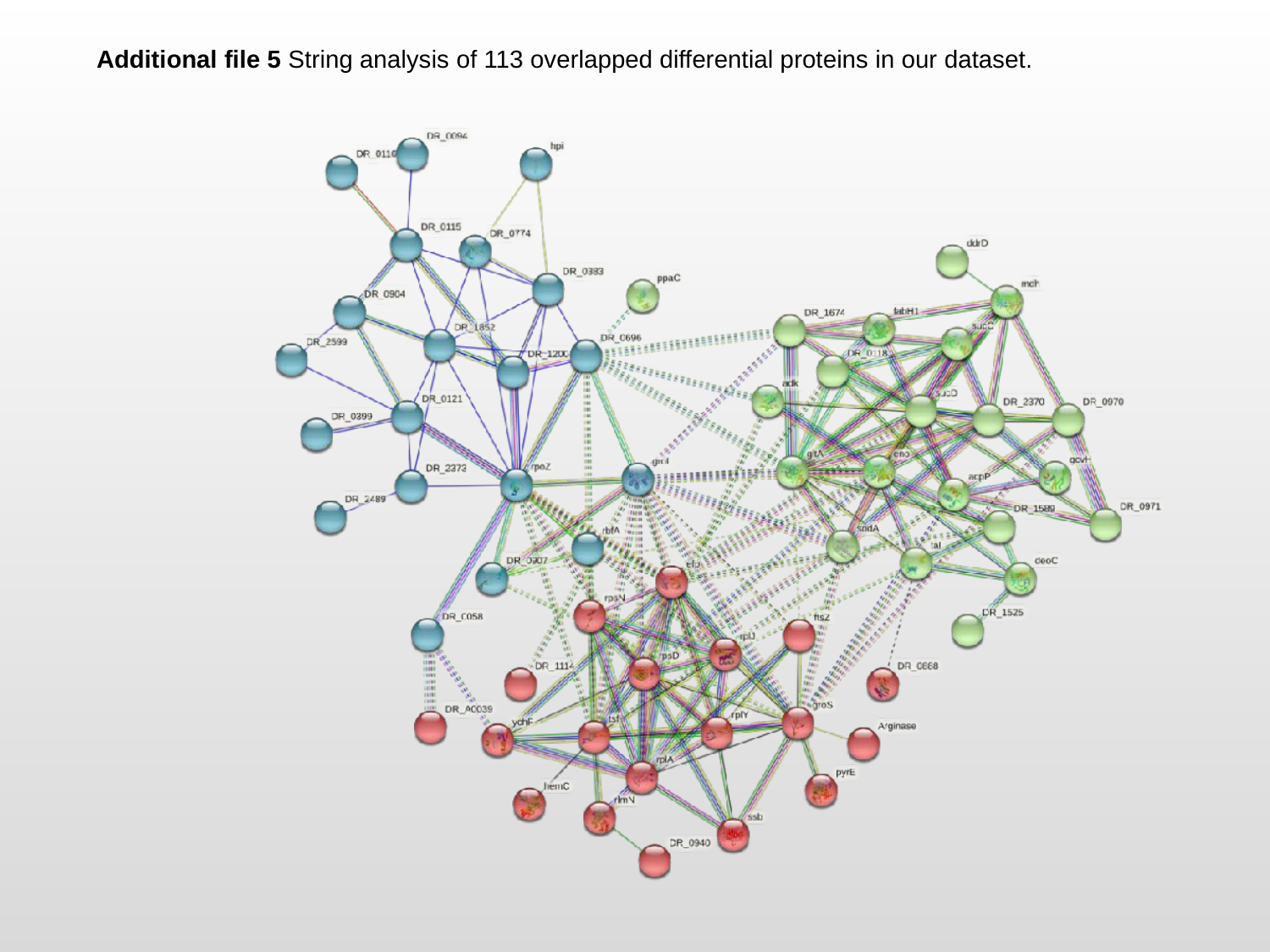

Additional file 5 String analysis of 113 overlapped differential proteins in our dataset.

Supplement: Supplementary file 5 — Additional file 5. String analysis of 113 overlapped differential proteins in our dataset. [file 12866_2022_2676_MOESM5_ESM.pptx]
