# Supplementary material for: iTRAQ-based proteomic analysis of Deinococcus radiodurans in response to 12C6+ heavy ion irradiation
Source: BMC Microbiol. 2022 Nov 4;22:264. doi: 10.1186/s12866-022-02676-x (PMC9635210; doi:10.1186/s12866-022-02676-x)
Supplement: Supplementary file 6 — Additional file 6. KEGG and GO enrichment analysis of 452 differential proteins in our dataset. [file 12866_2022_2676_MOESM6_ESM.pptx]

## Slide 1
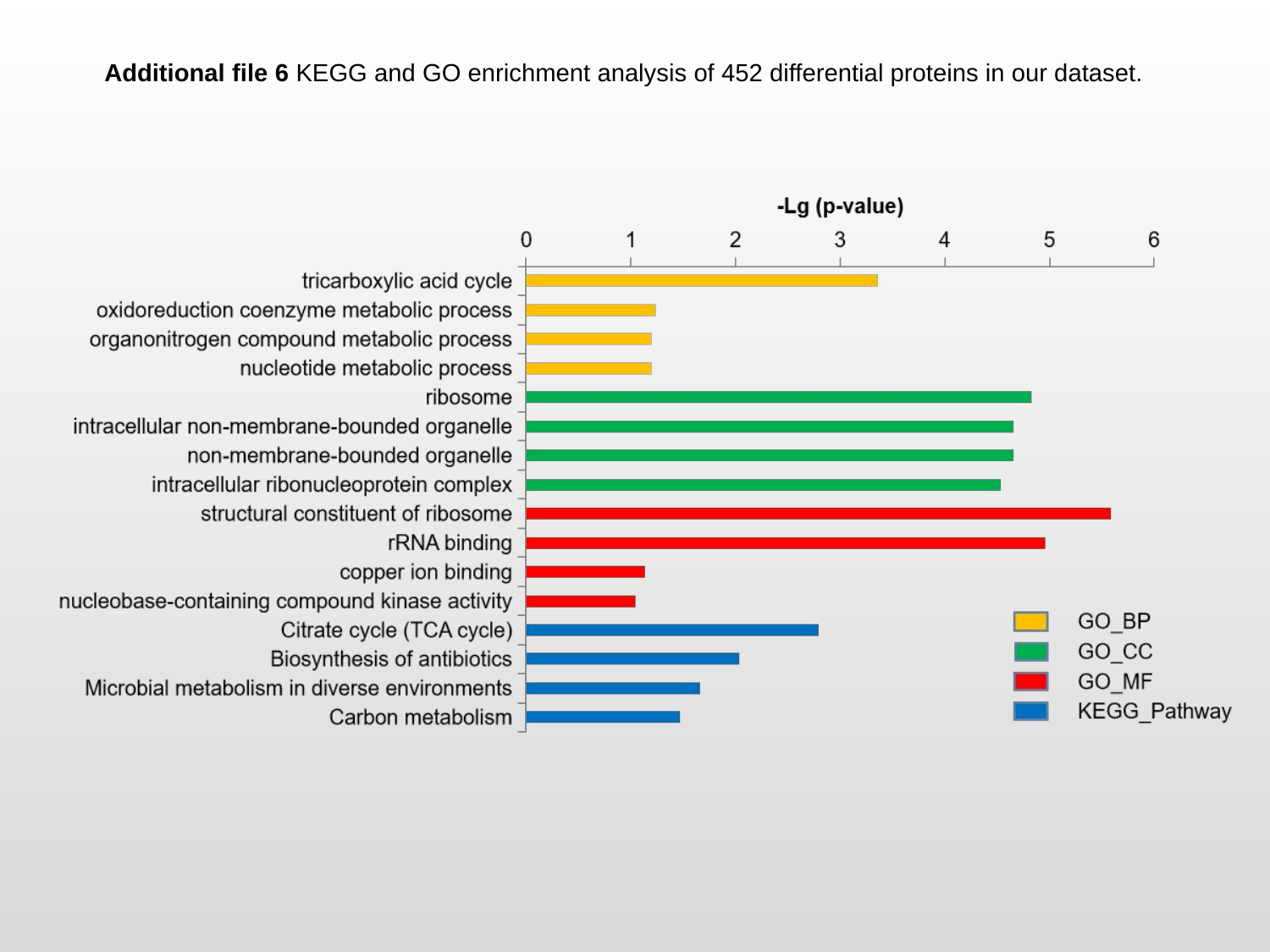

Additional file 6 KEGG and GO enrichment analysis of 452 differential proteins in our dataset.
