# Supplementary material for: iTRAQ-based proteomic analysis of Deinococcus radiodurans in response to 12C6+ heavy ion irradiation
Source: BMC Microbiol. 2022 Nov 4;22:264. doi: 10.1186/s12866-022-02676-x (PMC9635210; doi:10.1186/s12866-022-02676-x)
Supplement: Supplementary file 7 — Additional file 7: Supplementary file1. Preparation for iTRAQ quantitative proteomics study, related to additional file 1 [file 12866_2022_2676_MOESM7_ESM.pdf]

Supplementary file1 Preparation for iTRAQ quantitative proteomics study, related to additional file 1.

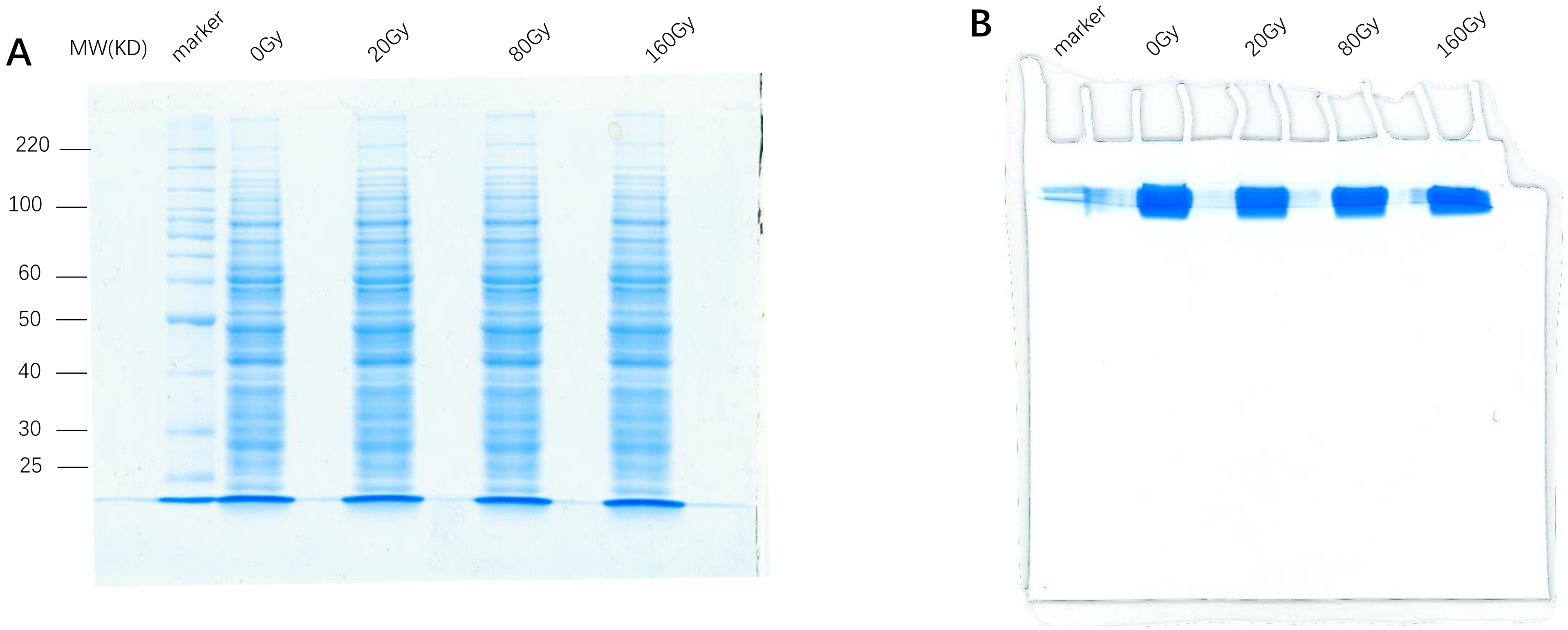

A, SDS-PAGE separation of whole cellular lysates of *D. radiodurans* treated with different dose of heavy ion irradiation.
B, Sample preparation for iTRAQ labeling.
